# Case Report: Multiple cutaneous keratoacanthoma-like lesions in a colorectal cancer patient treated with sintilimab

**DOI:** 10.3389/fimmu.2025.1535220

**Published:** 2025-03-03

**Authors:** Shiyi Li, Xianhui Ye, Xiaofen Li, Yu Yang

**Affiliations:** ^1^ Department of Medical Oncology, Cancer Center, West China Hospital, Sichuan University, Chengdu, China; ^2^ Division of Abdominal Tumor Multimodality Treatment, Cancer Center, West China Hospital, Sichuan University, Chengdu, China; ^3^ West China School of Public Health, Sichuan University, Chengdu, China; ^4^ Colorectal Cancer Center, West China Hospital, Sichuan University, Chengdu, China; ^5^ West China School of Nursing, Sichuan University, Chengdu, China

**Keywords:** sintilimab, keratoacanthoma, immunotherapy, adverse drug reaction, case report

## Abstract

Immune checkpoint inhibitors are increasingly being utilized for the treatment of advanced neoplastic disease, and Sintilimab as a selective anti–PD-1 antibody that inhibits interactions between PD-1 and its ligand, is a typical representative of them. Among all the adverse effects(AEs) of sintilimab, skin AEs had affected many people. Though exceedingly rare, eruptive keratoacanthomas-like lesion have been associated with the use of immune checkpoint inhibitors before. Here, we report a case of numerous eruptive keratoacanthoma-like lesions arising in a patient 2 weeks after initiation of sintilimab for rectal adenocarcinoma with liver metastasis. Although eruptive keratoacanthoma-like lesions secondary to sintilimab are exceptionally rarely reported, physicians should be aware of this cutaneous adverse effect as its use becomes more widespread.

## Introduction

Programmed cell death protein 1(PD-1) signaling suppresses T cell functions, including activation, proliferation, and cytokine production, cancer cells also use this pathway to escape immune surveillance (1). Sintilimab is a selective anti–PD-1 antibody that inhibits interactions between PD-1 and its ligand, PD-L1, which has potentially greater affinity against PD-1 than pembrolizumab or nivolumab as per the preclinical data (2), it is mainly used to treat cancers, including relapsed or refractory classic Hodgkin lymphoma, non-small cell lung cancer(NSCLC), liver cancer, and is also used to treat metastatic colorectal cancer. For the adverse effects(AEs) of sintilimab, in the official instructions, the AEs of sintilimab included pneumonia, diarrhea, colitis, hepatitis, nephritis, endocrinology diseases, skin AEs, infusion reactions, and other immune-related AEs. Some patients(>30%) who receive anti-PD1 therapy be affected by skin AEs. These predominantly manifest as eczema-like maculopapular rashes, lichenoid reactions, vitiligo-like lesions, or flares of psoriasis (3). Here, we report a case of a patient developing eruptive keratoacanthoma (K-A)-like lesions induced by treatment with sintilimab. Such eruptions represent an unusual cutaneous toxicity, which has so far been rarely reported.

## Case presentation

**Figure 1:**
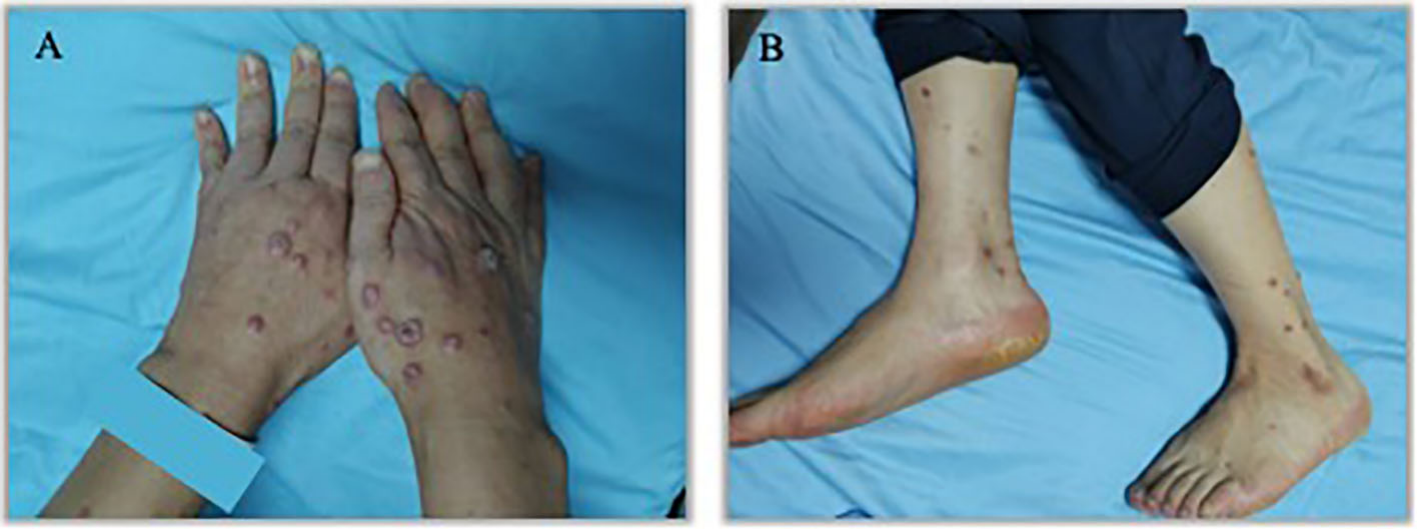
Initial presentation of eruptive keratoacanthomas on hands and lower legs occurring after the third treatment cycle of Sintilimab. **(A)**. K-A like lesions on hands **(B)**. K-A like lesions on lower legs.

**Figure 2:**
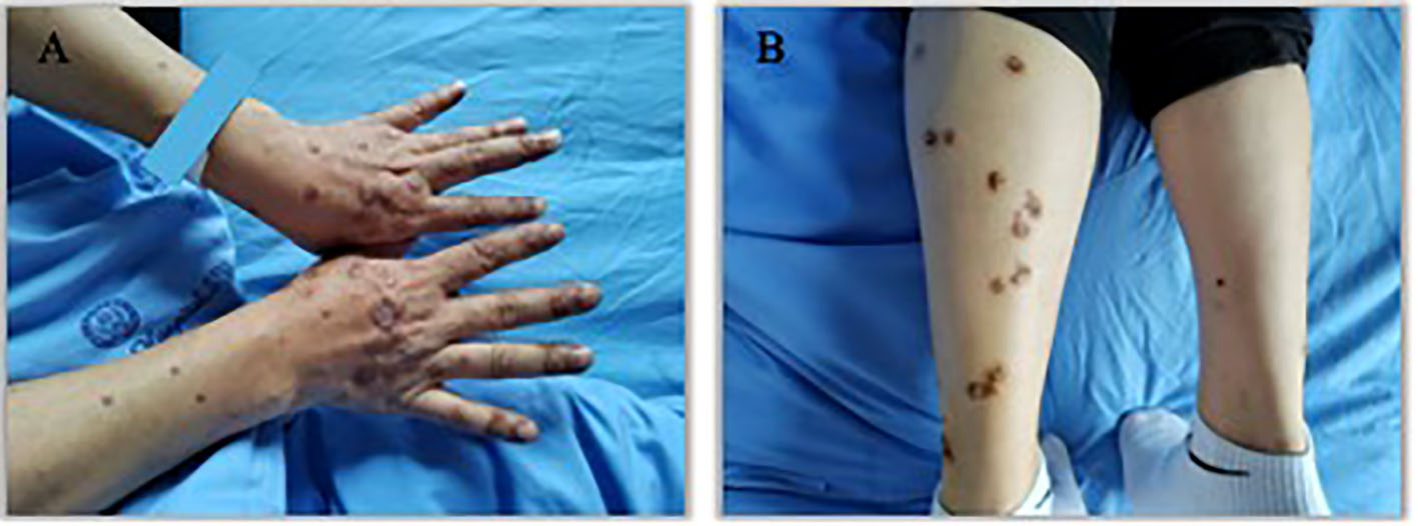
Appearance of hands and lower legs 1 month after the third treatment cycle of Sintilimab. **(A)**. K-A like lesions on hands 1 month after the third treatment **(B)**. K-A like lesions on lower legs 1 month after the third treatment.

**Figure 3:**
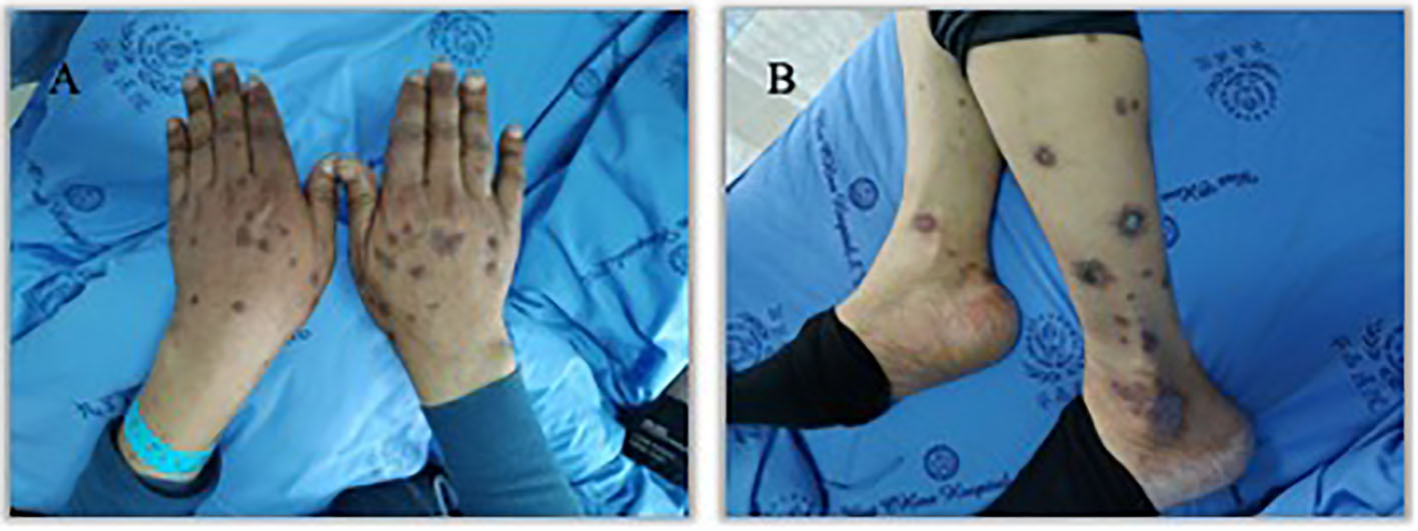
Appearance of hands and lower legs 3 month after the third treatment cycle of Sintilimab. **(A)**. K-A like lesions on hands 3 month after the third treatment **(B)**. K-A like lesions on lower legs 3 month after the third treatment.

**Figure 4:**
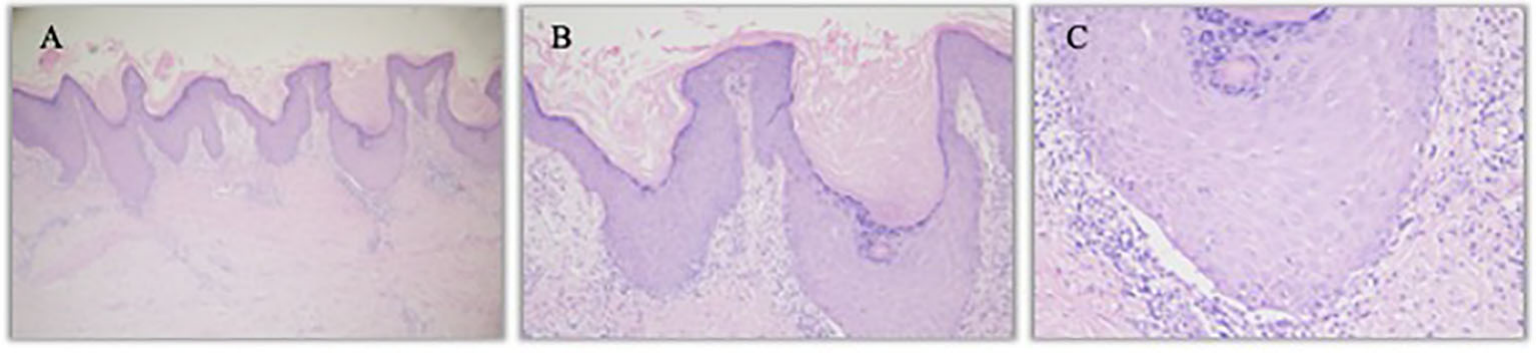
Hematoxylin- and eosin-stained image of left calf lesion. **(A)**. Left calf K-A like lesion (hematoxylin and eosin staining, 40×) **(B)**. Left calf epidermal hyperkeratosis with focal hypokeratosis (hematoxylin and eosin staining, 100×) **(C)**. Individual eosinophils infiltrate (hematoxylin and eosin staining, 200×).

An 57 years old man was treated with third-line sintilimab for rectal adenocarcinoma with liver metastasis, with the specific dose and medication pattern of 200mg intravenous drip every 3 weeks, and was also treated with oral regorafenib which starting from 4 months before sintilimab. Two weeks after the first treatment cycle, he developed sporadic skin lesions on both upper and lower limbs with progressive aggravation (**Figures 1**–**3**). The biopsy of skin lesions was performed, and the pathological findings of biopsy were as follows: left calf epidermal hyperkeratosis with focal hypokeratosis, hyperplasia of the acanthosis, liquefying degeneration of the basal layer, an equal number of lymphocytes and a few chromatophages in the superficial dermis, and individual eosinophils infiltrate (**Figure 4**). After three cycles of treatment with sintilimab, the efficacy was evaluated as progressive disease (PD), and then treatment of sintilimab was stopped. No special treatment was given to the skin lesions, and the lesions were gradually relieved after sintilimab withdrawal. At the 6-month follow-up, no recurrence or progression of skin lesions was observed.

## Discussion

We report a case of a patient developing eruptive keratoacanthoma (K-A)-like lesions, who treated with sintilimab and regorafenib starting 4 months before sintilimab. For the reason why regorafenib was ruled out as a contributing factor, the patient had also been treated with regorafenib 4 months before sintilimab, and showing no lesions associated with it.The patient developed K-A like lesions just 2 weeks after starting treatment with sintilimab, which is consistent with the common time range of skin lesions reported in previous studies due to immunotherapy (4), and the K-A like lesions was gradually relieved after sintilimab withdrawal. Besides, spontaneous K-A frequently presents as a solitary lesion, but multiple KAs may arise in the setting of immunomodulatory drug use (5). Therefore, we believe that the use of sintilimab caused the skin lesions in patient.

Immune checkpoints (ICs) are important immune regulators in maintaining immune homeostasis and preventing autoimmune diseases, while among all ICIs, the most studied ones are PD-1/PD-L1 antibodies (6). Sintilimab (Tyvyt^®^) is a fully human IgG4 monoclonal antibody that binds to PD-1, thereby blocking the interaction of PD-1 with its ligands (PD-L1 and PL-L2) and consequently helping to restore the endogenous antitumor T-cell response (7), and has become widespread in clinical practice in China. Withing the increasingly widespread use of ICI therapy in cancer treatment, a better understanding of immune-related adverse events (irAEs) is warranted, and both physicians and patients should be well-educated about these adverse events, though most of cutaneous adverse reactions are usually mild, some may still be life-threating (8). The mechanisms of ICI-induced cutaneous irAEs still remain unknown. According to recent studies, it is hypothesized that these adverse events may involve the activation of self-reactive T cells targeting common antigens, stimulation of B cells and humoral immunity, increased release of proinflammatory cytokines, and the occurrence of type IV hypersensitivity reactions, which have also suggested that the host’s genetic background may play a role in determining susceptibility to irAEs (9).

Keratoacanthoma (K-A) and squamous cell carcinoma (SCC) are rare side effects of programmed cell death ligand-1 (PD-L1) inhibitors that can disrupt therapy (4), it has rarely been reported in patients treated with Sindillizumab, we have compiled some reports on K-A like lesions caused by PD-1 inhibitors in recent years, listed in **Table 1**. The role of the immune system in the development and regression of K-A like lesions is not well understood, but some previous studies have reported that multiple immunosuppressive medications have been implicated in spontaneous K-A development (3) (10), suggesting that immune suppression may play a pathogenic role.

**Table 1:** A summary of previously reported cases of similar lesions due to PD-1 inhibitors.

No.	Author & Year	Disease	Treatment of disease	Lesions
1	Tembunde & Dika, 2023 (11)	Lung cancer	Pembrolizumab; 200 mg intravenous infusions every three weeks	The lesions started a week after beginning pembrolizumab for NSCLC, the lesion was intermittent, typically present for one to two weeks before resolving spontaneously.
2	Olsen et al, 2024 (10)	Recurrent metastatic oropharyngeal SCC^1^	Nivolumab	The lesions started after begin treatment with nivolumab 1 month, the patient was able to continue nivolumab infusions without significant interruption during the entire course of treatment for the KAs.
3	Fradet et al, 2019 (3)	Metastatic bronchial SCC^1^	Pembrolizumab	After treating for the second treatment cycle;developed hyper-keratotic nodular lesions (more than 30 lesions in total) on the dorsal region of his hands, knees and legs.
4	Crow et al, 2020 (12)	Metastatic melanoma	Pembrolizumab	Developed severely pruritic violaceous plaques on the trunk and extremities shortly after the initiation of pembrolizumab, concomitantly developed numerous scaly papules, plaques, and crateriform nodules on the extremities.
5	Crow et al, 2020 (12)	Metastatic mesothelioma	Pembrolizumab	Developed severely pruritic violaceous plaques on the trunk and extremities shortly after the initiation of pembrolizumab, concomitantly developed numerous scaly papules, plaques, and crateriform nodules on the extremities.
6	Crow et al, 2020 (12)	Metastatic lung adenocarcinoma	Nivolumab	Presented with pruritic, ill-defined scaly plaques on the trunk and extremities that were shown histopathologically to be spongiotic dermatitis, and numerous verrucous papules on the legs that were biopsy-proven KAs.

1, squamous cell carcinoma.

There are still some limitation in our study. Firstly, we cannot explain the mechanisms of ICI-induced cutaneous irAEs. Besides, more epidemiological data and evidence from experiments are needed to support the relationship we find between sintilimab and K-A like lesions. In the future, we will strive to collect more cases of immune-related skin adverse reactions and study their molecular mechanisms to provide references for clinical practice.

## Data Availability

The original contributions presented in the study are included in the article/supplementary material. Further inquiries can be directed to the corresponding authors.

## References

[1] Yi M Zheng X Niu M Zhu S Ge H Wu K. Combination strategies with PD-1/PD-L1 blockade: current advances and future directions. Mol Cancer. (2022) 21:1–3. doi: 10.1186/s12943-021-01489-2 PMC8780712 35062949

[2] Yang Y Wang Z Fang J Yu Q Han B Cang S. Efficacy and Safety of Sintilimab Plus Pemetrexed and Platinum as First-Line Treatment for Locally Advanced or Metastatic Nonsquamous NSCLC: a Randomized, Double-Blind, Phase 3 Study (Oncology pRogram by InnovENT anti-PD-1-11). J Thorac Oncol. (2020) 15:1636–46. doi: 10.1016/j.jtho.2020.07.014 32781263

[3] Fradet M Sibaud V Tournier E Lamant L Boulinguez S Brun A. Multiple keratoacanthoma-like lesions in a patient treated with pembrolizumab. Acta Dermato-Venereologica. (2019) 99:1301–2. doi: 10.2340/00015555-3301 31449315

[4] Poole M Schwartz RA Lambert WC Alhatem A. To treat or not to treat: PD-L1 inhibitor-induced keratoacanthoma and squamous cell carcinoma. Arch Dermatol Res. (2023) 315:903–15. doi: 10.1007/s00403-022-02468-3 36394634

[5] Kwiek B Schwartz RA. Keratoacanthoma (KA): An update and review. J Am Acad Dermatol. (2016) 74:1220–33. doi: 10.1016/j.jaad.2015.11.033 26853179

[6] He X Xu C. Immune checkpoint signaling and cancer immunotherapy. Cell Res. (2020) 30:660–9. doi: 10.1038/s41422-020-0343-4 PMC7395714 32467592

[7] Hoy SM. Sintilimab: first global approval. Drugs. (2019) 79:341–6. doi: 10.1007/s40265-019-1066-z 30742278

[8] Chen CH Yu HS Yu S. Cutaneous adverse events associated with immune checkpoint inhibitors: A review article. Curr Oncol. (2022) 29:2871–86. doi: 10.3390/curroncol29040234 PMC9032875 35448208

[9] Teng YS Yu S. Molecular mechanisms of cutaneous immune-related adverse events (irAEs) induced by immune checkpoint inhibitors. Curr Oncol. (2023) 30:6805–19. doi: 10.3390/curroncol30070498 PMC10378098 37504358

[10] Olsen E Svoboda SA Montanez-Wiscovich M Saikaly SK. Multiple eruptive keratoacanthomas secondary to nivolumab immunotherapy. J Immunother. (2024) 47:98–100. doi: 10.1097/CJI.0000000000000498 38009069

[11] Tembunde Y Dika MN. Pembrolizumab-induced eruptive keratoacanthomas and lichen planus in a lung cancer patient. Cureus. (2023) 15:e43402. doi: 10.7759/cureus.43402 37706118 PMC10496024

[12] Crow LD Perkins I Twigg AR Fassett MS LeBoit PE Berger TG. Treatment of PD-1/PD-L1 inhibitor-induced dermatitis resolves concomitant eruptive keratoacanthomas. JAMA Dermatol. (2020) 156:598–600. doi: 10.1001/jamadermatol.2020.0176 32211828

